# The effect of low-abundance OTU filtering methods on the reliability and variability of microbial composition assessed by 16S rRNA amplicon sequencing

**DOI:** 10.3389/fcimb.2023.1165295

**Published:** 2023-06-12

**Authors:** Maria Nikodemova, Elizabeth A. Holzhausen, Courtney L. Deblois, Jodi H. Barnet, Paul E. Peppard, Garret Suen, Kristen M. Malecki

**Affiliations:** ^1^ Population Health Sciences, School of Medicine and Public Health, University of Wisconsin-Madison, Madison, WI, United States; ^2^ Department of Physical Therapy, University of Florida, Gainesville, FL, United States; ^3^ Department of Integrative Physiology, University of Colorado Boulder, Boulder, CO, United States; ^4^ Department of Bacteriology, University of Wisconsin-Madison, Madison, WI, United States; ^5^ Microbiology Doctoral Training Program, University of Wisconsin-Madison, Madison, WI, United States; ^6^ Division of Environmental and Occupational Health Sciences, School of Public Health, University of Illinois at Chicago, Chicago, IL, United States

**Keywords:** OTU (Operational Taxonomic Unit), filtering, microbiome, low abundance, reliability – reproducibility of results, accuracy

## Abstract

PCR amplicon sequencing may lead to detection of spurious operational taxonomic units (OTUs), inflating estimates of gut microbial diversity. There is no consensus in the analytical approach as to what filtering methods should be applied to remove low-abundance OTUs; moreover, few studies have investigated the reliability of OTU detection within replicates. Here, we investigated the reliability of OTU detection (% agreement in detecting OTU in triplicates) and accuracy of their quantification (assessed by coefficient of variation (CV)) in human stool specimens. Stool samples were collected from 12 participants 22–55 years old. We applied several methods for filtering low-abundance OTUs and determined their impact on alpha-diversity and beta-diversity metrics. The reliability of OTU detection without any filtering was only 44.1% (SE=0.9) but increased after filtering low-abundance OTUs. After filtering OTUs with <0.1% abundance in the dataset, the reliability increased to 87.7% (SE=0.6) but at the expense of removing 6.97% reads from the dataset. When filtering was based on individual sample, the reliability increased to 73.1% after filtering OTUs with <10 copies while removing only 1.12% of reads. High abundance OTUs (>10 copies in sample) had lower CV, indicating better accuracy of quantification than low-abundance OTUs. Excluding very low-abundance OTUs had a significant impact on alpha-diversity metrics sensitive to the presence of rare species (observed OTUs, Chao1) but had little impact on relative abundance of major phyla and families and alpha-diversity metrics accounting for both richness and evenness (Shannon, Inverse Simpson). To increase the reliability of microbial composition, we advise removing OTUs with <10 copies in individual samples, particularly in studies where only one subsample per specimen is available for analysis.

## 1 Introduction

New technological advancements and computational methods have enabled investigations of microbial communities that are no longer limited to bacterial culturing methods, leading to the discovery of hundreds of new bacterial species in the human gut. Importantly, these studies highlight the significant role that the gut microbiome plays in both heath and disease. However, methodological variations in sample collection protocols, processing, and analytics make the reproducibility of findings across multiple studies or meta-analyses challenging (Lozupone et al., 2013; Goodrich et al., 2014). Currently, there is no consensus regarding best practices for human stool collection and analysis, leading to concerns regarding the reliability and reproducibility of these datasets. Several factors including DNA extraction methods, sequencing technologies, and analytical approaches are known to have a significant impact on the characterization of the stool microbiota (Lauber et al., 2010; Carroll et al., 2012; Goodrich et al., 2014; Sinha et al., 2015; Chiu and Chao, 2016; Sinha et al., 2017; Vogtmann et al., 2017; Antosca et al., 2020; Bartolomaeus et al., 2020), underscoring the importance of using standardized protocols to minimize bias.

In this study, we focused on analytical approaches to deal with low-abundance operational taxonomic units (OTUs) identified through 16S rRNA amplicon sequencing. These low-abundance OTUs, often thought to be spurious, can account for up to 50% of the detected OTUs in a sample, thereby skewing microbial diversity metrics (Eckburg et al., 2005; Gorzelak et al., 2015). The few studies that have examined the reliability (defined as % agreement in detecting OTU in replicates) of microbial composition in subsamples of the same fecal specimens showed that high abundance taxa are more reproducible compared to low-abundance taxa that are only sporadically detected within replicates (Wu et al., 2010; Gorzelak et al., 2015). Given that approximately 80% of bacterial species found in stool correspond to uncultivable species (Eckburg et al., 2005), estimating the microbiota composition relies on sequencing approaches. This is further compounded by PCR, which is used to generate amplicons and can lead to the overestimations of spurious OTUs, further inflating estimates of microbial diversity (Pienaar et al., 2006; Lahr and Katz, 2009; Goodrich et al., 2014; Potapov and Ong, 2017; Ma et al., 2019). One suggested approach to overcome these concerns is to sequence several replicates of the same specimen. While this approach can significantly improve the reliability of OTU detection (Zhou et al., 2008; Zhou et al., 2011), it is often not feasible particularly in studies with large numbers of subjects.

The aim of this study was to test if analyzing a single sample (oneplicate) per individual provides a reliable assessment of microbial composition. Reliability and validity are critical for advancing the use of human microbiome indicators in future human microbiome research and clinical trials. We investigated the reliability and variability of OTU detection within triplicates of the same human stool specimens. We applied several methods for filtering the low-abundance OTUs and determined their impact on alpha- and beta-diversity metrics. We provide an overview of comparative results and recommendations for data analysis that may improve the reliability and reproducibility of microbial composition estimates across studies.

## 2 Methods

### 2.1 Study participants and sample collection

The study was approved by the Health Sciences Institutional Review Board of the University of Wisconsin-Madison (#2016-0251). Recruitment and stool collection are described elsewhere (Holzhausen et al., 2021). Briefly, the study population consisted of 12 volunteers with a mean age of 35.4 years (SE = 3.1). Each stool specimen was aliquoted into three subsamples (triplicate) and frozen at −80°C within 30 min of stool production.

### 2.2 DNA extraction, PCR, and sequencing

DNA extraction from stool samples has been previously described in detail (Eggers et al., 2018; Eggers et al., 2019; Kates et al., 2020; Holzhausen et al., 2021). Briefly, DNA was extracted from lysed cells using phenol:chloroform:isoamylalcohol followed by isopropanol precipitation in the presence of sodium acetate. DNA was purified using a NucleoSpin Gel and PCR Clean-up Midi kit (Takara Bio USA, Inc., Mountain View, CA) as previously described (Kozich et al., 2013). Amplicons were generated from 25–50 ng of gDNA *via* PCR with primers (F- GTGCCAGCMGCCGCGGTAA; R- GGACTACHVGGGTWTCTAAT) targeting the V4 region (Kozich et al., 2013) along with sequencing adapters and barcodes to differentiate samples of the bacterial 16S rRNA gene. Amplicons were then subjected to gel electrophoresis on a 1% low melt agarose gel containing SYBR Safe DNA Gel Stain (Invitrogen). Bands of 380 bp were excised and purified using a Zymoclean DNA recovery kit (Zymo Research, Irvine, CA). DNA was sequenced on an Illumina MiSeq using a v2 2x250 bp paired-end sequencing kit (Illumina, San Diego, CA), with a final library concentration of 10 pmol/l and a 10% PhiX Control.

### 2.3 Data processing

Raw sequencing data were processed in mothur (version 1.43.0) following a Standard Operating Procedure for MiSeq data (Kozich et al., 2013). Briefly, contigs (overlapping sequences) were aligned to the SILVA (v132) database, reads of incorrect length were removed, and chimeras (determined by UCHIME) and undesirable reads (e.g., Archea, Eukaryota, chloroplasts, mitochondria, and unknowns) were removed. Sequences were assigned to OTUs with a threshold of 97% similarity using the GreenGenes (version gg_13_8_99) database (DeSantis et al., 2006; Edgar et al., 2011; Quast et al., 2013). Fastq files were submitted to NCBI’s Short Read Archive and are publicly available under accession number PRJNA962543.

### 2.4 Statistical analysis

Alpha-diversity was estimated using Observed OTUs, Chao 1, Shannon, and Inverse Simpson metrics (Shannon, 1948; Simpson, 1949; Chao, 1984), which were calculated using the Phyloseq package in R (McMurdie and Holmes, 2013). The precision of OTU quantification was analyzed by coefficient of variation (CV), which is calculated as the ratio of the standard deviation to the mean. A higher CV indicates greater dispersion in the variable. Means were calculated from copy counts in triplicate for each OTU. The effect of different filtering methods on relative sequence abundance was assessed by t-test followed by Mann–Whitney rank sum test. Differences were considered statistically significant at p < 0.05. Beta-diversity was estimated using Bray–Curtis dissimilarity, using the vegan package in R, and PERMANOVA tests were used to calculate proportion of variability explained by individual.

## 3 Results

### 3.1 Microbial composition of samples

Among the triplicate samples from 12 individuals, sequencing of the V4 region of the 16S rRNA gene resulted in 1,853,072 total raw reads or an average of 51,474 reads per sample. After filtering chimeras and removing low-quality reads and sequences of incorrect length, there were a total of 40,572 (SD = 10,883) reads per sample (range, 24,776–74,720). These included 3,761 unique OTUs, 80% of which were annotated at the genus level.

**Figure 1:**
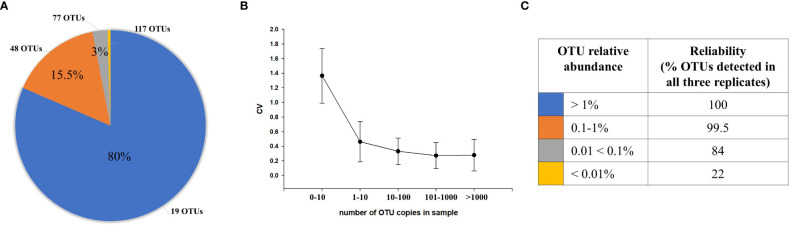
The distribution of OTUs and reliability of detection. **(A)** The average distribution of OTUs based on the relative sequence abundances within samples. The pie chart shows average number of OTUs in each relative abundance category (blue, >1%; orange, 0.1–1%; gray, 0.01<0.1%; yellow, <0.01%) and proportion (%) of sequences representing each relative abundance category On average, 19 OTUs with >1% relative abundance comprised 80% of all sequences within sample; 48 OTUs with 0.1%–1% relative abundance comprised 15.5% of sequences. The majority of OTUs (194) with <0.1% relative abundance (gray, yellow) comprised 4.5% of sequences. **(B)** Coefficient of variation (CV) calculated based on the number of copies for each OTU. **(C)** The reliability and accuracy of OTU detection. OTUs with higher relative sequence abundance were more reliably and accurately detected within replicates than lower abundance OTUs.

The distribution of OTUs based on relative abundance without filtering is shown in **Figure 1A**. The graph represents the mean (number of OTUs in each category) of all samples. On average, there were 266 unique OTUs per sample (**Figure 1A**). Of these, 19 (SE = 4.6) accounted for >80% of the total sequences (blue) with each OTU having a relative abundance >1%. For lower abundance OTUs, 48 (SE = 9.4) were detected between 0.1% and 1% and accounted for 15.5% of the reads (orange), 77 (SE = 19.4) OTUs had abundances between 0.01% and 0.1% (gray), and 117 OTUs had abundances <0.01% (yellow), accounting for ~3% of the total reads. Phylogenetic classification showed that the Firmicutes were the most abundant phyla (72.6%), followed by Bacteroidetes (18.6%), Verrucomicrobia (5.22%), Actinobacteria (2.7%), and Proteobacteria (0.61%).

### 3.2 Reliability and variability of OTU detection in sample replicates

A coefficient of variation (CV) was calculated for each OTU analyzed within triplicates of the same specimen to estimate the variability of their quantification (**Figure 1B**); in general, a lower CV indicates less dispersion and variability of quantification (Reed et al., 2002; Hanneman et al., 2011). We found that the CV for OTUs with >1,000 sequences in each replicate was <0.279; for 100–1,000 sequences, 0.273; for 10–100 sequences, 0.332; for 1–10 copies, 0.542; and for OTUs that were present in only one or two of the triplicates with <10 copies, 1.44.

We also found that OTUs with abundances >1%, having on average >371 sequences, were detected with 100% reliability (% OTUs detected in all three replicates) with good quantification accuracy (**Figure 1C**). OTUs with abundances between 0.1% and 1%, which had 37–371 sequences, were also detected with high reliability (99.5%) and accuracy. In contrast, reliability of detection were decreased (84%), while the variability in quantification increased for lower abundance OTUs detected between 0.01% and 0.1%, with an average number of sequences between 4 and 37. However, for OTUs with <0.01% abundance, which have <4 sequences on average, there was a lesser reliability (22%), and the variability in quantification was high (**Figure 1B**).

### 3.3 The effect of filtering methods on reliability of OTU detection

After assessing variability in the unfiltered sample, we then applied different approaches to filter low-abundance OTUs in order to determine which filtering method provided the best reliability of microbiome composition with respect to alpha-diversity metrics. The cutoff criteria for filtering OTUs were based on the OTU abundance: 1) in the whole dataset (sequences from all samples pooled together); 2) within an individual sample (each replicate was treated as an individual sample); or 3) within a triplicate (**Table 1**). Based on these cutoffs, removing low-abundance OTUs significantly increased the proportion of OTUs reliably detected across replicates from 44% (no OTUs removed) to up to 100% (OTUs filtered out if not present in all three replicates). Although low-abundance OTUs account for the largest proportion of unique OTUs, they represented only a small proportion of the sequences in the sample. Filtering methods excluded between 0.21% and 6.97% of the reads from the analysis (**Table 1**).

**Table 1:** The effect of filtering method on the reliability of OTU detection within triplicates.

	% OTUs in three replicates (SE)	% OTUs in two replicates (SE)	% OTUs in one replicate (SE)	% of reads filtered out (SE)
No filtering				
All OTUs included	44.1 (0.9)	15.6 (0.3)	40.2 (0.8)	0
Based on OTU abundance in whole dataset (pool of all samples) *				
OTU filtered if abundance <0.1% in dataset	87.7 (0.6)	4.3 (0.3)	8.1 (0.5)	6.97 (0.24)
OTU filtered if without >10 copies in at least one sample in dataset	70.3 (0.7)	13.6 (0.4)	14.6 (0.5)	0.88 (0.02)
Based on OTU abundance in individual sample **				
Filter OTUs with one copy	56.1 (0.9)	13.3 (0.3)	30.6 (0.8)	0.21 (0.01)
Filter OTUs with <10 copies	73.1 (0.8)	12 (0.5)	16.1 (0.5)	1.12 (0.03)
Based on OTU abundance in triplicates **				
Filter OTUs without ≥10 copies in at least one of the three replicates	96.8 (0.2)	2.2 (0.2)	0.9 (0.1)	0.88 (0.02)
Filter OTUs if not present in all three replicates	100 (0)	0	0	0.53 (0.02)

*OTUs fulfilling the filtering criteria were removed from all samples.

**OTUs were removed only from samples fulfilling the filtering criteria.

### 3.4 The effect of filtering methods on alpha-diversity metrics

Filtering low-abundance OTUs resulted in the removal of between 29% and 76% of the OTUs from the analysis with significant impact on alpha-diversity metrics (**Table 2**). As expected, the Chao1 estimator, which accounts for both the presence and abundance of OTUs and is known to be sensitive to communities with many species with low abundance (Kim et al., 2017), was significantly affected by all filtering methods. Other alpha-diversity metrics including Shannon’s and Inverse Simpson’s, which account for both richness and evenness, were less affected by low-abundance OTU removal. Filtering OTUs with <0.1% abundance in the dataset had the most significant impact on all alpha-diversity metrics. Most filtering methods did not have significant effects on the relative abundance of the five major phyla (**Table 3**); however, excluding OTUs with <0.1% abundance in the dataset significantly impacted phyla with smaller relative abundances, such as Actinobacteria and Proteobacteria.

**Table 2:** The effect of filtering method on different α-diversity metrics.

Filtering method	ObservedOTUsMean (SE)	Chao1Mean (SE)	ShannonMean (SE)	IInverse SimpsonMean (SE)
No filtering				
All OTUs included	266 (4.5)	380.9 (7.6)	3.243 (0.02)	13.289 (0.49)
Based on OTU abundance in whole dataset				
Filter OTUs with <0.1% abundance in dataset	65 (0.6)^**^	67.9 (0.7)^**^	2.922 (0.03)^**^	11.299 (0.38)^*^
Filter OTUs without >10 reads in at least one sample in dataset	189 (2.1)^**^	219.0 (2.6)^**^	3.218 (0.03)	13.196 (0.49)
Based on OTU abundance in individual sample				
Filter OTUs with one read	188 (2.7)^**^	188 (2.7)^**^	3.2 (0.03)	13.2 (0.5)
Filter OTUs with < 10 reads	106 (1.3)^**^	106 (1.3)^**^	3.164 (0.03)^*^	12.955 (0.48)
Based on OTU abundance in triplicate				
Filter OTUs without ≥10 reads in at least one of the three replicates	121 (1.4)^**^	121.4 (1.4)^**^	3.180 (0.03)	13.029 (0.48)
Filter OTUs if not present in all three replicates	169 (2.2)^**^	182.2 (2.6)^**^	3.206 (0.03)	13.141 (0.49)

*p<0.05 vs All OTUs included, **p<0.001 vs All OTUs included.

**Table 3:** The effects of filtering method on the relative sequence abundance of the top 5 phyla.

Filtering method	Firmicutes % (SE)	Bacteroidetes % (SE)	Verrucomicrobia % (SE)	Actinobacteria % (SE)	Proteobacteria % (SE)
Based on abundance in whole dataset					
All OTUs included	72.6 (0.8)	18.6 (0.7)	5.22 (0.6)	2.70 (0.2)	0.610 (0.03)
Filter out OTUs with <0.1% abundance in dataset	72.5 (0.8)	18.9 (0.7)	5.72 (0.7)	2.48* (0.2)	0.367** (0.04)
Filter out OTUs without count >10 in at least one sample in dataset	72.6 (0.8)	18.6 (0.7)	5.24 (0.6)	2.70 (0.2)	0.599 (0.04)
Based on abundance in individual sample					
Filter out OTUs with one read	72.6 (0.8)	18.6 (0.7)	5.23 (0.62)	2.70 (0.2)	0.60 (0.04)
Filter out OTUs with count ≤ 10	72.5 (0.8)	18.7 (0.7)	5.27 (0.6)	2.68 (0.2)	0.564 (0.04)
Based on abundance in triplicate					
Filter out OTUs without count >10 in at least one of the three replicates	72.6 (0.8)	18.7 (0.7)	5.26 (0.6)	2.68 (0.02)	0.571 (0.04)
Filter out OTUs if not present in all three replicates	72.6 (0.8)	18.6 (0.7)	5.24 (0.6)	2.70 (0.02)	0.574 (0.04)

*p < 0.05 vs. all OTUs included.

**p < 0.001 vs. all OTUs included.

### 3.5 The effect of filtering methods on beta-diversity

**Figure 2:**
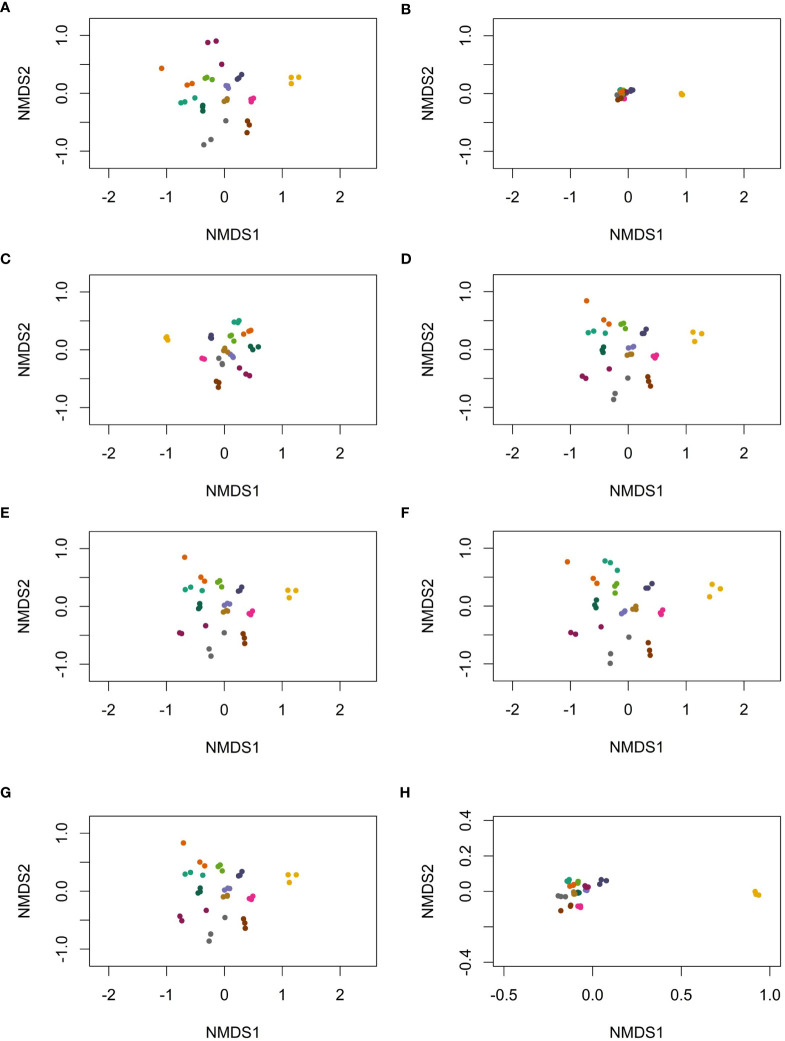
The beta-diversity, based on Bray–Curtis dissimilarity for various filtering methods, including **(A)** no filtering, all OTUs included, **(B)** OTUs with relative abundance <0.1% removed, **(C)** OTUs with >10 copies in at least one sample in the dataset removed, **(D)** OTUs with one copy filtered from individual replicates, **(E)** OTUs with <10 copies filtered from individual replicates, **(F)** OTUs with <10 copies in at least one of three replicates filtered from all replicates, **(G)** OTUs not present in all three replicates filtered, and **(H)** a zoomed-in plot **(B)**, where OTUs with relative abundance <0.1% were filtered. Each color represents an individual; three dots of the same color represent three replicates from the individual.

Filtering low-abundance OTUs resulted in tighter clustering between points (reduced BC dissimilarity) compared to the unfiltered dataset (**Figure 2**). As expected, filtering based on overall relative abundance <0.1% (**Figures 2B, H**) resulted in the most dramatic reduction in BC dissimilarity between samples. Despite tighter clustering, PERMANOVA analyses revealed that the proportion of variability attributable to an individual (i.e., R^2^) was 0.94 (p = 0.001), regardless of the filtering method. This suggests that all filtering methods were able to reduce variation in overall microbiome signatures, without losing important biological signals such as the individual providing the sample.

## 4 Discussion

While gut microbiome research has significantly expanded over the last decade, there are no broadly accepted consensus protocols that would ensure the reproducibility and reliability of study outcomes. The Microbiome Quality Control (MBQC) project and other initiatives have identified methodological differences in sample collection, storage conditions, DNA extraction methods, sequencing technologies, and data analysis as key sources of variability among studies that may conceal or dilute assessment of true biological effects (Goodrich et al., 2014; Sinha et al., 2015; Sinha et al., 2017). The estimates of microbiome composition that rely on PCR and sequencing technologies are sources of methodological artifacts that may lead to the detection of spurious OTUs (Acinas et al., 2005; Sipos et al., 2007; Aird et al., 2011; Goodrich et al., 2014) and thus complicate downstream analyses given their overwhelming contributions to the diversity of a sample (Ley et al., 2008; Goodrich et al., 2014). However, to distinguish spurious and rare OTUs is challenging, especially in situations where only one subsample per subject is analyzed. To circumvent this problem, low-abundance OTUs are often excluded from the analysis. However, filtering approaches vary among studies, and there is no agreed-upon consensus as to what filtering threshold should be employed.

To address the methodological question of how best to filter and the implications of filtering in study outcome measures, we sequenced three replicates from 12 human specimens to determine the reliability of OTU detection. Only 44% of the OTUs were shared by all three replicates, and over 40% of the OTUs were found only in one of the replicates. OTUs that were detected sporadically within triplicates had very low abundances, usually with <10 copies, and had low accuracy of quantification, as assessed by CV. In contrast, OTUs with >0.1% abundance within a sample were reliably detected with good accuracy of quantification. Overall, our data indicate that OTUs with lesser abundance had greater CVs, suggesting lower detection accuracy. The CV is commonly used to determine intra- and inter-assay variability (Reed et al., 2002) to assess pipetting techniques, user effect, batch effect, and lab to lab variability. In such studies, a CV < 0.1 for intra-assay variability and CV <0.15 for inter-assay variability is considered excellent. Although the same scale may not be applicable to evaluate the accuracy of OTU detection by 16S sequencing because the sources of quantification errors are different, the increasing CV values with decreasing OTU abundance provides better understanding of limitation of low-abundance OTU detection by 16S sequencing.

Amplicon sequencing of soil samples found even smaller OTU overlap in three replicates (8.2%), suggesting low reproducibility of amplicon-sequencing-based microbiome composition (Zhou et al., 2011). Others have attributed such low reproducibility to several factors, one of them being non-representative subsampling (Zhou et al., 2008). Beyond analytic variability, some variability may also be due to inadequate mixing of fecal sample before taking subsamples for DNA extraction that are not representative and would disproportionally affect rare taxa.

In general, the approaches we used to filter low-abundance OTUs improved the reliability of microbiota composition estimates, but there are several factors to consider when choosing the appropriate filtering methods. We found that methods based on OTU abundance in the whole dataset (e.g., pooling all sequences from all samples) provided good reliability (% OTUs detected in all three replicates), but they are affected by study sample size, and thus, the threshold for OTU removal may differ among studies. For example, when filtering OTUs with <0.1% abundance, the threshold is 10,000 copies for a dataset with 10,000,000 sequences but only 1,000 copies in smaller dataset with 1,000,000 sequences. Therefore, the microbiota composition estimates of the same sample will be different in different datasets.

Removing OTUs based on the number of copies within individual samples also improved the reliability of replicates while retaining higher proportions of rare OTUs. This method is independent of sample size and would thus be more consistent from study to study. Our data suggest that OTUs with <10 copies in the samples are less reliably and accurately detected and are randomly distributed within triplicates. Therefore, it is reasonable to remove these OTUs from analyses.

Filtering methods significantly affected beta-diversity. Removing OTUs based on the copy number in individual samples retained relatively larger beta-diversity compared to filtering by relative abundance in the dataset. The distance and relative position in the Bray–Curtis dissimilarity plot among individuals are dependent on the filtering method, further underlying the significance of analytical approach, which may contribute to low reproducibility and reliability of gut microglial studies. Although the most optimal filtering method would depend on overall study goals, based on our analysis, we would recommend censoring OTUs with <10 copies in individual samples during analysis of 16S rRNA gene sequencing. However, the best reliability of microbiota composition estimates is achieved when at least two replicates of the same specimen are analyzed; this approach would be recommended when low-abundance OTUs are important to consider.

The study has several strengths in that it analyzes repeated human microbiome samples and considers numerous ways of filtering and comparison of results. While sample size could be considered a limitation of this study, the analysis of 12 individuals with three replicates provided sample size with sufficient statistical power for the analyses. While many consider whole genome sequencing (WGS) the gold standard alternative to 16s sequencing, WGS has other analytic challenges and can be very cost prohibitive. The use of 16s amplicon sequencing is not suitable to annotate taxa at the species level, which is a limitation. However, it is a cost-effective method and will likely remain to be widely used to annotate the microbiome.

## 5 Conclusions

The aim of the study was to evaluate the reliability of OTU detection and to assess the impact of different filtering methods on microbiome alpha- and beta-diversity and composition. To increase the reliability of microbial composition, we advise removing OTUs with <10 copies in individual samples, particularly in studies where only one subsample per specimen is available for analysis. Excluding very low-abundance OTUs has a significant impact on alpha-diversity metrics sensitive to the presence of rare species but had little impact on relative abundance of major phyla and families and alpha-diversity metrics accounting for both richness and evenness.

## Data availability statement

The datasets presented in this study can be found in online repositories. The names of the repository/repositories and accession number(s) can be found below: https://www.ncbi.nlm.nih.gov/, PRJNA962543.

## Ethics statement

The studies involving human participants were reviewed and approved by Health Sciences Institutional Review Board of the University of Wisconsin - Madison (#2016-0251). Written informed consent for participation was not required for this study in accordance with the national legislation and the institutional requirements.

## Author contributions

MN conceived the study. MN and EH designed the study and collected the samples. PP, KM and GS supervised this work. LD conducted DNA extraction, PCR, and sequencing. JB provided statistical consultation. EH and MN drafted the paper. All authors contributed to the article and approved the submitted version.

## References

[B1] Acinas S. G. Sarma-Rupavtarm R. Klepac-Ceraj V. Polz M. F. (2005). PCR-induced sequence artifacts and bias: insights from comparison of two 16S rRNA clone libraries constructed from the same sample. Appl. Environ. Microbiol. 71, 8966–8969. doi: 10.1128/AEM.71.12.8966-8969.2005 16332901 PMC1317340

[B2] Aird D. Ross M. G. Chen W. S. Danielsson M. Fennell T. Russ C.. (2011). Analyzing and minimizing PCR amplification bias in illumina sequencing libraries. Genome Biol. 12, R18. doi: 10.1186/gb-2011-12-2-r18 21338519 PMC3188800

[B3] Antosca K. Hoen A. G. Palys T. Hilliard M. Morrison H. G. Coker M.. (2020). Reliability of stool microbiome methods for DNA yields and sequencing among infants and young children. Microbiologyopen 9, e1018. doi: 10.1002/mbo3.1018 32166902 PMC7221451

[B4] Bartolomaeus T. U. P. Birkner T. Bartolomaeus H. Lober U. Avery E. G. Mahler A.. (2020). Quantifying technical confounders in microbiome studies. Cardiovasc. Res 117(3):863–875. doi: 10.1093/cvr/cvaa128 32374853

[B5] Carroll I. M. Ringel-Kulka T. Siddle J. P. Klaenhammer T. R. Ringel Y. (2012). Characterization of the fecal microbiota using high-throughput sequencing reveals a stable microbial community during storage. PloS One 7, e46953. doi: 10.1371/journal.pone.0046953 23071673 PMC3465312

[B6] Chao A. (1984). Nonparametric estimation of the number of classes in a population. Scandinavian J. Stat 11, 265–270.

[B7] Chiu C. H. Chao A. (2016). Estimating and comparing microbial diversity in the presence of sequencing errors. PeerJ 4, e1634. doi: 10.7717/peerj.1634 26855872 PMC4741086

[B8] DeSantis T. Z. Hugenholtz P. Larsen N. Rojas M. Brodie E. L. Keller K.. (2006). Greengenes, a chimera-checked 16S rRNA gene database and workbench compatible with ARB. Appl. Environ. Microbiol. 72, 5069–5072. doi: 10.1128/AEM.03006-05 16820507 PMC1489311

[B9] Eckburg P. B. Bik E. M. Bernstein C. N. Purdom E. Dethlefsen L. Sargent M.. (2005). Diversity of the human intestinal microbial flora. Science 308, 1635–1638. doi: 10.1126/science.1110591 15831718 PMC1395357

[B10] Edgar R. C. Haas B. J. Clemente J. C. Quince C. Knight R. (2011). UCHIME improves sensitivity and speed of chimera detection. Bioinformatics 27, 2194–2200. doi: 10.1093/bioinformatics/btr381 21700674 PMC3150044

[B11] Eggers S. Malecki K. M. Peppard P. Mares J. Shirley D. Shukla S. K.. (2018). Wisconsin Microbiome study, a cross-sectional investigation of dietary fibre, microbiome composition and antibiotic-resistant organisms: rationale and methods. BMJ Open 8, e019450. doi: 10.1136/bmjopen-2017-019450 PMC5875638 29588324

[B12] Eggers S. Safdar N. Sethi A. K. Suen G. Peppard P. E. Kates A. E.. (2019). Urinary lead concentration and composition of the adult gut microbiota in a cross-sectional population-based sample. Environ. Int. 133, 105122. doi: 10.1016/j.envint.2019.105122 31518933 PMC7230144

[B13] Goodrich J. K. Di Rienzi S. C. Poole A. C. Koren O. Walters W. A. Caporaso J. G.. (2014). Conducting a microbiome study. Cell 158, 250–262. doi: 10.1016/j.cell.2014.06.037 25036628 PMC5074386

[B14] Gorzelak M. A. Gill S. K. Tasnim N. Ahmadi-Vand Z. Jay M. Gibson D. L. (2015). Methods for improving human gut microbiome data by reducing variability through sample processing and storage of stool. PloS One 10, e0134802. doi: 10.1371/journal.pone.0134802 26252519 PMC4529225

[B15] Hanneman S. K. Cox C. D. Green K. E. Kang D. H. (2011). Estimating intra- and inter-assay v ariability in salivary cortisol. Biol. Res. Nurs. 13, 243–250. doi: 10.1177/1099800411404061 21498487

[B16] Holzhausen E. A. Nikodemova M. Deblois C. L. Barnet J. H. Peppard P. E. Suen G. (2021). Et. al. assessing the impact of storage time on the stability of stool microbiota richness, diversity, and composition. Gut Pathog. 13, 75. doi: 10.1186/s13099-021-00470-0 34930464 PMC8686582

[B17] Kates A. E. Jarrett O. Skarlupka J. H. Sethi A. Duster M. Watson L.. (2020). Household pet ownership and the microbial diversity of the human gut microbiota. Front. Cell Infect. Microbiol. 10, 73. doi: 10.3389/fcimb.2020.00073 32185142 PMC7058978

[B18] Kim B. R. Shin J. Guevarra R. Lee J. H. Kim D. W. Seol K. H.. (2017). Deciphering diversity indices for a better understanding of microbial communities. J. Microbiol. Biotechnol. 27, 2089–2093. doi: 10.4014/jmb.1709.09027 29032640

[B19] Kozich J. J. Westcott S. L. Baxter N. T. Highlander S. K. Schloss P. D. (2013). Development of a dual-index sequencing strategy and curation pipeline for analyzing amplicon sequence data on the MiSeq illumina sequencing platform. Appl. Environ. Microbiol. 79, 5112–5120. doi: 10.1128/AEM.01043-13 23793624 PMC3753973

[B20] Lahr D. J. Katz L. A. (2009). Reducing the impact of PCR-mediated recombination in molecular evolution and environmental studies using a new-generation high-fidelity DNA polymerase. Biotechniques 47, 857–866. doi: 10.2144/000113219 19852769

[B21] Lauber C. L. Zhou N. Gordon J. I. Knight R. Fierer N. (2010). Effect of storage conditions on the assessment of bacterial community structure in soil and human-associated samples. FEMS Microbiol. Lett. 307, 80–86. doi: 10.1111/j.1574-6968.2010.01965.x 20412303 PMC3148093

[B22] Ley R. E. Hamady M. Lozupone C. Turnbaugh P. J. Ramey R. R. Bircher J. S.. (2008). Evolution of mammals and their gut microbes. Science 320, 1647–1651. doi: 10.1126/science.1155725 18497261 PMC2649005

[B23] Lozupone C. A. Stombaugh J. Gonzalez A. Ackermann G. Wendel D. Vázquez-Baeza Y.. (2013). Meta-analyses of studies of the human microbiota. Genome Res. 23, 1704–1714. doi: 10.1101/gr.151803.112 23861384 PMC3787266

[B24] Ma X. Shao Y. Tian L. Flasch D. A. Mulder H. L. Edmonson M. N.. (2019). Analysis of error profiles in deep next-generation sequencing data. Genome Biol. 20, 50. doi: 10.1186/s13059-019-1659-6 30867008 PMC6417284

[B25] McMurdie P. J. Holmes S. (2013). Phyloseq: an r package for reproducible interactive analysis a nd graphics of microbiome census data. PloS One 8, e61217. doi: 10.1371/journal.pone.0061217 23630581 PMC3632530

[B26] Pienaar E. Theron M. Nelson M. Viljoen H. J. (2006). A quantitative model of error accumulation during PCR amplification. Comput. Biol. Chem. 30, 102–111. doi: 10.1016/j.compbiolchem.2005.11.002 16412692 PMC1544370

[B27] Potapov V. Ong J. L. (2017). Examining sources of error in PCR by single-molecule sequencing. PloS One 12, e0169774. doi: 10.1371/journal.pone.0169774 28060945 PMC5218489

[B28] Quast C. Pruesse E. Yilmaz P. Gerken J. Schweer T. Yarza P.. (2013). The SILVA ribosomal RNA gene database project: improved data processing and web-based tools. Nucleic Acids Res. 41, D590–D596. doi: 10.1093/nar/gks1219 23193283 PMC3531112

[B29] Reed G. F. Lynn F. Meade B. D. (2002). Use of coefficient of variation in assessing variability of quantitative assays. Clin. Diagn. Lab. Immunol. 9, 1235–1239. doi: 10.1128/CDLI.9.6.1235-1239.2002 12414755 PMC130103

[B30] Shannon C. E. (1948). A mathematicl theory of communication. Bell System Tech. J. 27, 379–423. doi: 10.1002/j.1538-7305.1948.tb01338.x

[B31] Simpson E. H. (1949). Measurement of species diversity. Nature 163, 688. doi: 10.1038/163688a0

[B32] Sinha R. Abnet C. C. White O. Knight R. Huttenhower C. (2015). The microbiome quality control project: baseline study design and future directions. Genome Biol. 16, 276. doi: 10.1186/s13059-015-0841-8 26653756 PMC4674991

[B33] Sinha R. Abu-Ali G. Vogtmann E. Fodor A. A. Ren B. Amir A.. (2017). Assessment of variation in microbial community amplicon sequencing by the microbiome quality control (MBQC) project consortium. Nat. Biotechnol. 35, 1077–1086. doi: 10.1038/nbt.3981 28967885 PMC5839636

[B34] Sipos R. Székely A. J. Palatinszky M. Révész S. Márialigeti K. Nikolausz M. (2007). Effect of primer mismatch, annealing temperature and PCR cycle number on 16S rRNA gene-targetting bacterial community analysis. FEMS Microbiol. Ecol. 60, 341–350. doi: 10.1111/j.1574-6941.2007.00283.x 17343679

[B35] Vogtmann E. Chen J. Amir A. Shi J. Abnet C. C. Nelson H.. (2017). Comparison of collection methods for fecal samples in microbiome studies. Am. J. Epidemiol. 185, 115–123. doi: 10.1093/aje/kww177 27986704 PMC5253972

[B36] Wu G. D. Lewis J. D. Hoffmann C. Chen Y. Y. Knight R. Bittinger K.. (2010). Sampling and pyrosequencing methods for characterizing bacterial communities in the human gut using 16S sequence tags. BMC Microbiol. 10, 206. doi: 10.1186/1471-2180-10-206 20673359 PMC2921404

[B37] Zhou J. Kang S. Schadt C. W. (2008). And garten CT, jr. spatial scaling of functional gene diversity across various microbial taxa. Proc. Natl. Acad. Sci. U.S.A. 105, 7768–7773. doi: 10.1073/pnas.0709016105 18509054 PMC2409408

[B38] Zhou J. Wu L. Deng Y. Zhi X. Jiang Y. H. Tu Q.. (2011). Reproducibility and quantitation of amplicon sequencing-based detection. Isme J. 5, 1303–1313. doi: 10.1038/ismej.2011.11 21346791 PMC3146266

