# Corrigendum: The effect of low-abundance OTU filtering methods on the reliability and variability of microbial composition assessed by 16S rRNA amplicon sequencing

**DOI:** 10.3389/fcimb.2024.1419131

**Published:** 2024-06-13

**Authors:** Maria Nikodemova, Elizabeth A. Holzhausen, Courtney L. Deblois, Jodi H. Barnet, Paul E. Peppard, Garret Suen, Kristen M. Malecki

**Affiliations:** ^1^ Population Health Sciences, School of Medicine and Public Health, University of Wisconsin-Madison, Madison, WI, United States; ^2^ Department of Physical Therapy, University of Florida, Gainesville, FL, United States; ^3^ Department of Integrative Physiology, University of Colorado Boulder, Boulder, CO, United States; ^4^ Department of Bacteriology, University of Wisconsin-Madison, Madison, WI, United States; ^5^ Microbiology Doctoral Training Program, University of Wisconsin-Madison, Madison, WI, United States; ^6^ Division of Environmental and Occupational Health Sciences, School of Public Health, University of Illinois at Chicago, Chicago, IL, United States

**Keywords:** OTU (Operational Taxonomic Unit), filtering, microbiome, low abundance, reliability – reproducibility of results, accuracy

## Error in Figure/Table

In the published article, there was an error in **Table 2** as published. Table 1 was incorrectly duplicated, while **Table 2** was omitted. The corrected **Table 2** and its caption The effect of filtering method on difference α-diversity metrics appear below.

**Table 2:** The effect of filtering method on different α-diversity metrics.

Filtering method	Observed OTUs Mean (SE)	Chao1 Mean (SE)	Shannon Mean (SE)	Inverse Simpson Mean (SE)
No filtering				
All OTUs included	266 (4.5)	380.9 (7.6)	3.243 (0.02)	13.289 (0.49)
Based on OTU abundance in whole dataset				
Filter OTUs with <0.1% abundance in dataset	65 (0.6)^**^	67.9 (0.7)^**^	2.922 (0.03)^**^	11.299 (0.38)^*^
Filter OTUs without >10 reads in at least one sample in dataset	189 (2.1)^**^	219.0 (2.6)^**^	3.218 (0.03)	13.196 (0.49)
Based on OTU abundance in individual sample				
Filter OTUs with one read	188 (2.7)^**^	188 (2.7)^**^	3.2 (0.03)	13.2 (0.5)
Filter OTUs with < 10 reads	106 (1.3)^**^	106 (1.3)^**^	3.164 (0.03)^*^	12.955 (0.48)
Based on OTU abundance in triplicate				
Filter OTUs without ≥10 reads in at least one of the three replicates	121 (1.4)^**^	121.4 (1.4)^**^	3.180 (0.03)	13.029 (0.48)
Filter OTUs if not present in all three replicates	169 (2.2)^**^	182.2 (2.6)^**^	3.206 (0.03)	13.141 (0.49)

*p<0.05 vs All OTUs included, **p<0.001 vs All OTUs included.

The authors apologize for this error and state that this does not change the scientific conclusions of the article in any way. The original article has been updated.

